# Probing the Interaction Forces of Prostate Cancer Cells with Collagen I and Bone Marrow Derived Stem Cells on the Single Cell Level

**DOI:** 10.1371/journal.pone.0057706

**Published:** 2013-03-05

**Authors:** Ediz Sariisik, Denitsa Docheva, Daniela Padula, Cvetan Popov, Jan Opfer, Matthias Schieker, Hauke Clausen-Schaumann, Martin Benoit

**Affiliations:** 1 Chair of Biophysics and New Materials, Ludwig-Maximilians-University, Munich, Germany; 2 Center for NanoScience, Ludwig-Maximilians-University, Munich, Germany; 3 Experimental Surgery and Regenerative Medicine, Department of Surgery, Ludwig-Maximilians-University, Munich, Germany; 4 Department of Applied Sciences and Mechatronics, Munich University of Applied Sciences, Munich, Germany; 5 Center for Applied Tissue Engineering and Regenerative Medicine Munich University of Applied Sciences, Munich, Germany; Swiss Federal Institute of Technology Zurich, Switzerland

## Abstract

Adhesion of metastasizing prostate carcinoma cells was quantified for two carcinoma model cell lines LNCaP (lymph node-specific) and PC3 (bone marrow-specific). By time-lapse microscopy and force spectroscopy we found PC3 cells to preferentially adhere to bone marrow-derived mesenchymal stem cells (SCP1 cell line). Using atomic force microscopy (AFM) based force spectroscopy, the mechanical pattern of the adhesion to SCP1 cells was characterized for both prostate cancer cell lines and compared to a substrate consisting of pure collagen type I. PC3 cells dissipated more energy (27.6 aJ) during the forced de-adhesion AFM experiments and showed significantly more adhesive and stronger bonds compared to LNCaP cells (20.1 aJ). The characteristic signatures of the detachment force traces revealed that, in contrast to the LNCaP cells, PC3 cells seem to utilize their filopodia in addition to establish adhesive bonds. Taken together, our study clearly demonstrates that PC3 cells have a superior adhesive affinity to bone marrow mesenchymal stem cells, compared to LNCaP. Semi-quantitative PCR on both prostate carcinoma cell lines revealed the expression of two Col-I binding integrin receptors, α1β1 and α2β1 in PC3 cells, suggesting their possible involvement in the specific interaction to the substrates. Further understanding of the exact mechanisms behind this phenomenon might lead to optimized therapeutic applications targeting the metastatic behavior of certain prostate cancer cells towards bone tissue.

## Introduction

Prostate cancer is one of the most common malignancies and a leading cause of cancer death among men in Europe. Almost all patients with advanced prostate cancer show metastasis in bone, which is often the only detectable site of the cancer spread [1]. Furthermore, the prostate cancer in bone is frequently diagnosed before detection of the primary disease and once the prostate cancer cells are engrafted into the skeleton, curative therapy is no longer possible and palliative treatment becomes the only option [2]. Although researchers are now beginning to understand the mechanisms of cancer growth in bone, the initial steps of tumour cell-to-bone interactions that promote the expansion of the metastatic deposit is not yet fully understood. Hence, there is clearly a need to elucidate the factors underlying the spreading of prostate cancer particularly to the skeleton.

It has been suggested that cancer metastasis in bone is the result of a complex interplay between prostate cancer cells with the bone matrix proteins and with the cell types residing in the bone tissue such as osteoblasts and osteoclasts[3]–[5]. We and others have demonstrated that the prostate cancer cell line PC3, isolated from the bone marrow, has a significantly higher adhesion to the major bone protein collagen type I (Col-I) than the prostate adenocarcinoma cell line LNCaP which derives from a non-bone metastatic site [6], [7]. These results suggest that affinity to Col-I might be one of the molecular factors contributing to the progression of some prostate cancer cells into the bone.

With regards to the cellular factors, apart from osteoblasts and osteoclasts, another intriguing participant that has been recently reported is the cell population residing in the bone marrow, termed mesenchymal stem cells (MSC). MSCs are the early progenitors of osteoblasts and they can be further expanded and differentiated into specialized mesenchymal cells such as adipocytes, chondrocytes, or osteoblasts in vitro [8]. Cross et al., 2007, have suggested that MSCs may play a major role in supporting prostate cancer growth and survival in the bone [9].

From the initial establishment to the later expansion in the bone, the prostate cancer cells require invasive capability. Nabha et al., 2008 found that MSCs stimulated the invasive ability of PC3 cells through Col-I by inducing the secretion of the protease MMP-12 from PC3 cells [10]. In addition, a recent article demonstrated that mesenchymal fibroblasts can lead the collective cancer invasion by remodelling their surrounding matrix, and thus creating physical space through which the cancer cells can simply follow [11].

These data already suggest specific cross-talk between prostate cancer cells and MSCs, but still it is not clear whether and how strong these two cell types can interact and what could be the mechanisms behind this interaction. Specific molecules on the cell surface can mediate cellular interactions. Such molecular interactions have been measured mechanically by tracing the force required to separate receptor-ligand pairs or interacting cells with optical tweezers, the biomembrane force probe or atomic force microscopy [12]–[14]. Such experiments are not only able to measure molecular detachment events but also to probe the mechanical embedding and anchoring of the measured molecules in the cells [15]–[17]. Thus, the main aim of this study was to gain new insights into prostate cancer cell interactions with MSCs with an emphasis on the mechanical forces occurring on the molecular level. In particular, the quantification of the adhesive forces between prostate cancer cells and the matrix protein Col-I appeared to be essential, because previous studies investigating the affinity of prostate cancer cells to various matrix proteins did not determine their interaction force.

As prostate cancer cells, we used PC3 cells, which have originated from bone marrow metastasis and as controls, LNCaP cells, which were isolated from lymph node metastasis. As mesenchymal stem cells we used an immortalized MSC cell line named SCP1 [18], which possesses the typical MSC features, such as self-renewal and multipotency and allows for long-term standardized analysis. We first visualized the adhesion and propagation rate of prostate cancer cells on MSC monolayers by time lapse fluorescence microscopy on the multicellular level. Then, we characterized the actual physical forces involved in single cell-to-substrate contacts by force microscopy with an AFM: both prostate cancer cells lines were immobilized on an AFM cantilever and brought into contact with a Col-I coated substrate. Finally, we measured cell-to-cell adhesive forces between PC3 or LNCaP prostate cancer cells, attached to an AFM cantilever, and a mesenchymal stem cell (SCP1) monolayer.

## Materials and Methods

### Cell Culture for Time-lapse Microscopy

PC3 (derived from bone metastasis) and LNCaP cells (derived from lymph node metastasis) were obtained from ATCC (Wesel, Germany). PC3 cells were maintained in RPMI-1640 cell culture media (PAA, Cölbe, Germany) and 10% FBS (Sigma-Aldrich, Munich, Germany). The SCP1 cell line is an immortalized human mesenchymal stem cell line fully described in Böker et al. 2008 [18]. LNCaP and SCP1 cells were cultured in MEM Alpha GlutaMAX culture media (Invitrogen, Karlsruhe, Germany) supplemented with 10% FBS. During routine cell culture, all cell types were grown up to 80% confluence in T-25 or T-75 culture flasks and maintained at 37°C in 5% humidified CO_2_. The culture medium was changed three times per week and for cell passaging, cells were detached by treatment with 1x trypsin/EDTA solution (PAA). The preparation of the cells prior to AFM measurements is described bellow in the paragraph “Cell capture”.

### Time-lapse Microscopy and Quantification of Cell Adhesion

**Figure 1:**
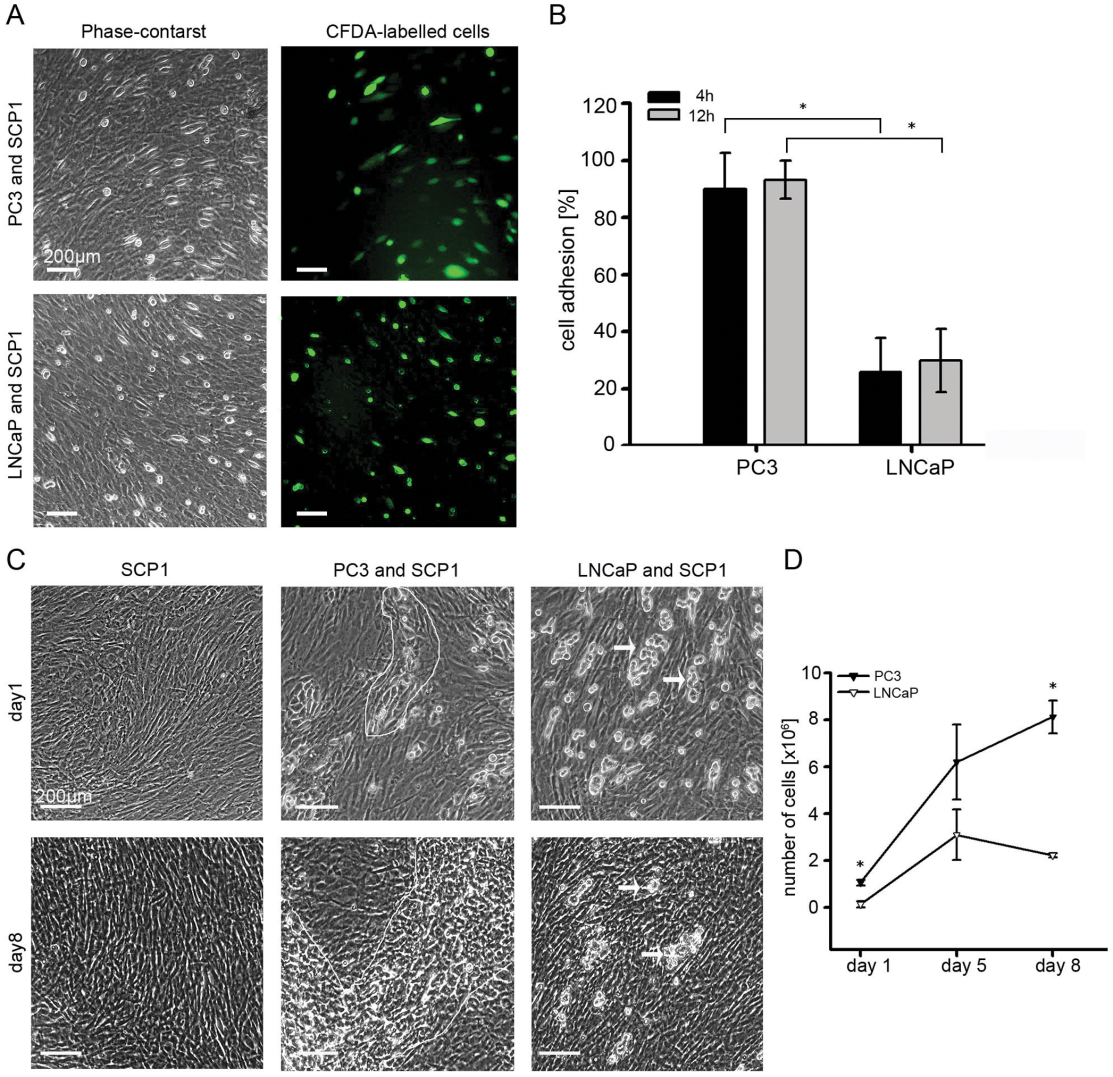
Cell adhesion and expansion of PC3 and LNCaP cells on SCP1 monolayers. (**A**) Phase-contrast and fluorescent microscopy of CFDA-labelled PC3 and LNCaP cells plated on SCP1 monolayers in 6-well dishes. Images are taken after 4 h. (**B**) Quantification of adherent PC3 and LNCaP cells after 4 and 12 h cultivation on SCP1 monolayers. The percentage of adherent cells was quantified first, by manual counting of the CFDA-labelled cells with the cell counter tool in Image J software and second, by comparing to the initial number of plated cells (5×10^5^ cells/well). In the images also a slight background of CFDA dye particles is visible (more apparent in the LNCaP image). The analyses revealed that already at 4 h PC3 cells completely adhered on SPC1 cells while LNCaP cells had a significantly lower adhesion rate at 4 h and 12 h. The graph bars show mean ± SD of four independent experiments (p<0.0001, unpaired t-test). (**C**) PC and LNCaP cells (2×10^5^ cells/well) were grown on SCP1 monolayers in 6-well dishes for up to 8 days. Phase-contrast images demonstrated the formation and propagation of PC3 colonies (outlined) on the top of SCP1 cells between day 1 and 8. In contrast, LNCaP cells formed small cell clusters (arrows) that did not expand but rather regressed by day 8. (**D**) Quantification of PC and LNCaP cell numbers after 1, 5 and 8 days of cultivation on SCP1 monolayers. The proliferation of PC3 and LNCaP cells was calculated by subtracting the SCP1 control monolayers from the total cell count of the co-culture. Similarly to the microscopy data, the quantitative analysis confirmed that PC3 cells but not LNCaP were able to divide and further expand on SCP1 cells. The graph shows mean ± SD of three independent experiments for each time point (p<0.0001, unpaired t-test).

SCP1 cells (10^6^ cells) were grown in 6-well dishes to full confluence (as shown in **Fig. 1C**). PC3 and LNCaP cells were labelled with the 10 µM green fluorescent CFDA dye (carboxyfluorescein diacetate, acetoxymethyl ester, Invitrogen) and then plated on the formed SCP1 monolayers (5×10^5^ cancer cells/well). Directly after, microscopy images were collected with 25 minutes intervals for at least 12 hours. During this time the cells were kept in a bio-chamber, providing stable 37°C and 5% humidified CO2 atmosphere (Pecon, Erbach, Germnay), mounted on an inverted optical microscope (Axiovert 100, Carl Zeiss Hallbergmoos, Germany). The images were taken with an AxioCam MRm CCD camera (Carl Zeiss) and by using manually the cell counter tool of Image J version1.40 software (National Institute of Health, USA) the number of adherent cells was estimated and shown as percentage to the initial cell input at 4 and 12 hours.

### Cell Proliferation Analysis

SCP1 monolayers were formed as described above and 2×10^5^ PC3 and LNCaP cells were added and left to expand onto SPC1 cells for a period of 8 days. In addition, several culture wells were retained only with SCP1 cells (*SCP1_mono_*) in order to be used as controls for the quantification analysis. The co-cultures (*PC3_+SCP1_, LNCaP_+SCP1_*) were monitored microscopically and photographed with the AxioCam MRm camera (Zeiss). At day 1, 5 and 8 the co-cultures were trypsinized and by using Neubauer cell counting chamber, the total cell number was estimated. The proliferation of PC3 and LNCaP cells on SCP1 monolayer (*PC3_on mono_*, *LNCaP_on mono_*) was calculated as follows:
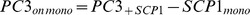


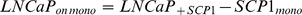



### Immunocytochemistry

Prior to protein coating, glass slides were cleaned with 70% ethanol and then autoclaved. In order to verify the collagen type I (Col-I) -coating of the glass slides and the collagen I expression on SCP1, slides and SCP1 monolayers were prepared as follows. SCP1 cells were grown on glass slides for two days in order to form confluent cell monolayers, while Col-I - coated glass slides were prepared by adding 1 mg/ml Col-I solution at 4°C overnight. Next, SCP1 monolayers and the Col-I-coated slides were fixed with pure acetone for 20 min at -20°C, rinsed with PBS. Image-iT FX Signal Enhancer (an Invitrogen product for background reduction and signal intensification of Alexa Flour secondary antibodies) was applied for 30 min and blocked with 10% BSA for 1 hour. The primary mouse monoclonal anti-collagen-I antibody (Sigma) was applied overnight at 4°C. This step was followed by incubation with the secondary anti-mouse antibody conjugated to Alexa Flour 488 for 1 hour and the nuclear stain DAPI for 5 minutes. In parallel, negative controls were carried out by omitting the primary antibody. Photomicrographs were taken with an Axiocam MRm camera on an Axioskope 2 microscope (Carl Zeiss) using 40x objective.

### AFM Setup

Force Spectroscopy experiments were conducted using a NanoWizard II together with a CellHesion module (JPK Instuments, Berlin, Germany), mounted on a Zeiss Axiovert 200 M (Carl Zeiss, Goettingen, Germany) with a custom made temperature unit for 37°C. For reduced influence of ambient noise, the Axiovert was placed on an active isolation table (Micro 60, Halcyonics, Göttingen, Germany) against vibrations and the whole setup was placed into a 1 m^3^ soundproof box also stabilizing the temperature of the entire experiment.

The force sensors used for force spectroscopy were tipless silicon nitride cantilevers with a nominal spring constant of 0.01 N/m (Tipless, MLCT-O10, Veeco, USA). These force sensors with a low spring constant turned out to be most suited for cell adhesion measurements. In particular, the tipless plane surface provides an adhesion area for a single cell. By coating this surface with cell adhesives, such as lectins (e. g. concavalin A) or positively charged polymers (e.g. polylysine) various cell types can be firmly and fast attached to the sensor [16], [19], [20]. Prostate cancer cells turned out to stably adhere to lysine electrostatically and furthermore keep their spherical shape throughout the entire measurement process rather than spreading as on Col-I coated surfaces. Prior to cell adhesion spectroscopy experiments, the force sensors therefore were coated with Poly D-Lysine (PDL, Millipore, USA) in a solution of 100 µg/ml PDL overnight. PDL was used instead of PLL because it is less degradable and the cells did not tend to spread. The spring constants of the force sensors were determined individually by the thermal noise method [21].

Force-distance curves were recorded while the piezo traveled in a closed loop up to 20 µm at an approach velocity of 7 µm/s until a trigger force of 100pN was reached, and a retraction velocity of 3 µm/s.

### Substrate Preparations

**Figure 2:**
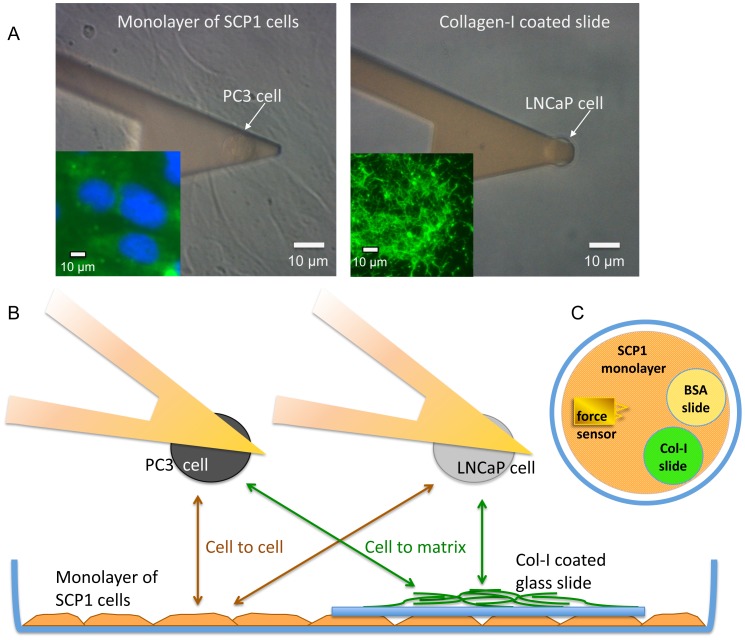
Schematic representation of the experimental setup. (**A**) Phase contrast images of a prostate cancer cell attached to the cantilever (arrows) above an SCP1 monolayer (left) and a Col-I-coated slide (right). The scale bars indicate 10 µm. On the lower left corners immunofluorescence images are inserted. Col-I, labeled with AlexaFluor488 fluorescence dye appears in green and cell nuclei, stained with DAPI in blue. (**B**) Single cells from two different prostate cancer cell lines (PC3 and LNCaP) were immobilized to a tipless AFM cantilever (force sensor) in order to study their interaction forces with the apical surface of a SCP-1 monolayer (representing mesenchymal stem cells) or with Col-I (representing bone matrix). (C) Schematic top view of the culture dish lid with a BSA-coated glass cover slip (as substrate for fishing a gently injected prostate cancer cell) and a Col-I coated glass cover slip both on top of a monolayer of mesenchymal stem cells. For calibration and fishing a cell, the force sensor visits the BSA slide, for the experiment on collagen the Col-I slide and for the experiment on mesenchymal cells the SCP1 monolayer.

We have used collagen type-I (Col-I)-coated glass cover slips and SCP1 monolayers as substrates for the AFM force spectroscopy experiments within the same culture dish lid. To form SCP1 monolayers, SCP1 cells were grown on untreated culture dish lids (petri dish 35×10 mm, nunc A/S, Roskilde, Denmark) for two days at 37°C, 5% CO_2_. Prior to use, they were washed with and covered by 1.5 ml fresh serum-free MEM-Alpha medium (Invitrogen, Karlsruhe, Germany) supplemented with 15 mM Hepes (Sigma-Aldrich, Germany) resulting in a CO_2_ independent measurement medium. For cell to Col-I measurements glass cover slips (Ø 15 mm washed in 70% ethanol and distilled water) were coated with Col-I (100 µg/ml) at 4°C overnight. Prior to the cell adhesion measurements, the Col-I-coated cover slips were placed on top of the SCP1 monolayer in the culture dish lids (as depicted in **Fig. 2B**). An additional glass cover slip coated with BSA (0.5%w/v) at 4°C overnight was placed on top of another section of the SCP1 monolayer and it was used for cell capture (see next section). The culture dish lid, containing all three types of substrates (BSA, Col-I and SCP-1 monolayer) was then mounted on a temperature-controlled stage in the AFM and it was left to equilibrate for 10 min in ambient air at 37°C.

### Cell Culture and Cell Capture for Force Spectroscopy

Cells (LNCaP or PC3) grown to 80% confluency were incubated in trypsin/EDTA solution (0.02%) for 5 min to 10 min until released from the substrate after washing with PBS lacking calcium and magnesium. This procedure should remove any matrix proteins possibly covering the cell surfaces without affecting the integrin receptors [22], [23]. Then the cells were transferred with additional MEM-Alpha medium into a centrifuge tube. The cells were then spun down (1000 rpm, 3 min) before resuspending the pellet with fresh MEM-Alpha medium. The cells were left in an incubator at 37°C for 15 min., in order to adapt them to the measurement temperature of 37°C in the AFM.

Either PC3 or LNCaP cells (approx. 2 µ*l* containing 100 to 300 cells) were then gently injected onto the non-adhesive BSA-coated cover slip in order to subsequently capture one of them with the adhesive PDL-coated cantilever: The adhesive cantilever was positioned over one of the obviously healthy cells (medium size, round shaped at normal contrast, no blebs, no other abnormal indications in shape) on the BSA-coated cover slip, and lowered in a stepwise manner until it was close to the surface of this cell. Then, the cantilever was gently in held contact with the cell for a few seconds before the cantilever-bound cell was lifted vertically by approximately 100 µm [24]. The cell was allowed to establish firm adhesion on the cantilever for a couple of minutes. Some cells (approx. 10%) refused to adhere firmly to the lever rather hanging loosely as determined by gently shaking the microscope and watching the cell move with the induced agitation. In this case the cell was washed off the cantilever by lifting it out of the liquid and back again in order to capture a new cell. In the case of firm adhesion, the cell was used for adhesion experiments and monitored by the experimenter via the light microscope image during the entire period of measurements.

### Cell Adhesion Force Measurements

The cell immobilized on the force sensor was pushed against either the SCP1 monolayer or the Col-I-coated slide with a contact force of 100 pN. The contact time between the probe cell and its substrate was set to 0s resulting in a forced contact of effectively 0.3 s in order to limit adhesion rates (percentage of curves with adhesive events) to a range as low as possible. Adhesion rates below 30% provide a high probability of detecting single molecular interactions [24]. At higher adhesion rates individual unbinding steps tend to result from multiple molecular bonds acting in parallel. To quantify the differences between cell lines and surfaces, this short contact time and low contact force of 100pN was applied throughout the entire experiments. The retraction velocity was set to 3 µm/s as a compromise between hydrodynamic drag, which increases with velocity (here at about 5 pN) and thermal drift effects which decrease with velocity. The retraction distance was set to 20 µm to account for long tethers (**Fig. 2A**) and to assure the cell had separated from the substrate completely after each cycle. Force measurements on the SCP1 monolayer and Col-I were performed within the same culture dish, whereas for each cell immobilized on a cantilever a new dish was prepared. This resulted in two experiments per culture dish: Either a LNCaP cell on the cantilever vs. the Col-I-coated glass and subsequently vs. the apical surface area of the SCP1 monolayer or a PC3 cell on the cantilever vs. Col-I and then vs. SCP1 monolayer **(** **Fig. 2B** **)**. The order of the substrates was also reversed within the PC3 experiments, showing identical results. In each experiment (probing one type of cell to one type of substrate) at least 80 force curves were taken between one cell on the cantilever and one substrate type. Altogether, at least 10 independent experiments for each combination of cell-substrate interactions (LNCaP vs. Col-I; LNCaP vs. SCP1; PC3 vs. Col-I; PC3 vs. SCP1) were carried out, yielding at least 800 force curves per class of interaction. During the entire experiment, we used the optical microscope to monitor the spherical shape and the firm attachment of the cell immobilized to the PDL-coated force sensor **(** **Fig. 2A** **)**. Furthermore, we proved by prolonged cell contacts of 1 min at 500 pN to Col-I that the cell immobilized to the force sensor can sustain adhesion forces of at least 8.5 nN without detaching from the sensor.

### Cell Adhesion Force Evaluation

**Figure 3:**
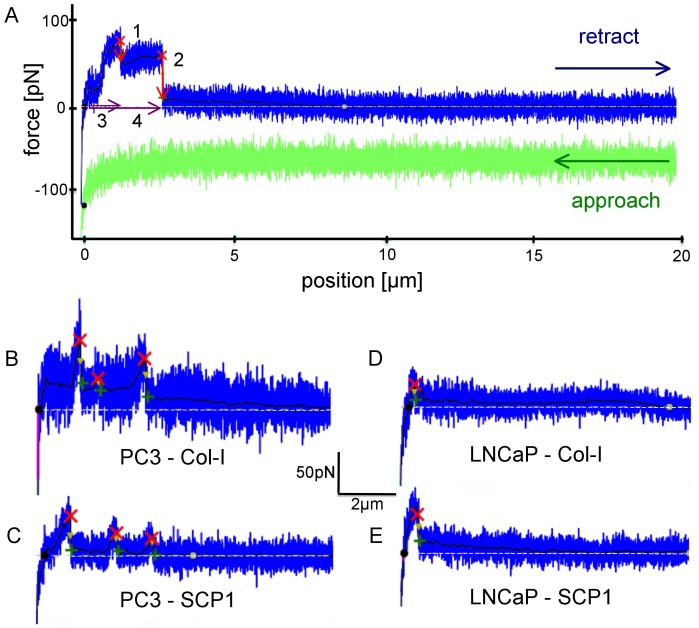
Representative force-distance curves: in green the approach of the force sensor with a prostate cancer cell to the substrate and in blue the retraction (for clarity the blue curve is shifted by approximately 50 pN with respect to the green curve). The lowest data point to the left marks the contact force of 100 pN; the white dotted line represents the baseline intersecting the retrace curve at the black circle defining the cell surface; the black line is the de-noised signal and the red crosses indicate detected de-adhesion steps where the adhesion force evaluation takes place. (**A**) Force curve from a PC3-cell interacting with Col-I for illustrating the adhesion force evaluation: Red arrow #1: step height of the first de-adhesion event in the retraction curve. The detachment force is the absolute measure from the red cross down to the base line; #2: step height of the second de-adhesion event after a force plateau of 0.9 µm in length; #3: step position of the first de-adhesion event; #4: step position of the second de-adhesion event. (For definitions see **Cell Adhesion Force Evaluation** in the Materials and Methods section). Characteristic curves from each of the four different types of experiments are represented: (**B**) PC3 on Col-I, (**C**) PC3 on SCP1 monolayer, (**D**) LNCaP on Col-I and (**E**) LNCaP on SCP1 monolayer.

For data analysis only the retraction parts of the approach-retract cycles were evaluated. In order to obtain characteristic quantitative information from the force-distance curves, a custom-designed data evaluation and step detection software [25] was used to denoise the signal (black lines in **Fig. 3**), find the baseline (dashed lines in **Fig. 3**), correct for hydrodynamic drag and possible drift and to extract the following parameters:

- **step height** [pN] describing the difference in force measured before and after an individual detachment event, visible as a force step. The algorithm identifies such a step by maxima in the derivative of the denoised signal that surmount a certain threshold and marks it by a small red cross (cf. also **Fig. 3**). The last step in a force curve is the most reliable one since in contrast to all other (intermediate) steps no other connection between cell and substrate persists. Therefore the last step is not potentially diminished by other bonds existing in parallel.
- **adhesion rate** [%] describing the fraction of curves with at least one detected force step.
- **number of steps** describing the average number of steps detected per curve (only counting curves with at least one detected force step).
- **step position** [µm] describing the distance between the contact point (black circle at the intersection of baseline and retrace curve) and a force step.
- **work of detachment** [aJ] describing the energy dissipated during that force experiment by integrating the area between baseline (zero force) and retract curve. (Note: this has no trivial relation to the adhesion energy. In fact, velocity dependent viscous and plastic deformation of the cell and the cell membrane itself strongly contribute to the work of detachment far from the thermodynamic equilibrium).
- **detachment force** [pN] describing the highest measured adhesion (global maximum) per curve.
- **peak position** [nm] describing the distance between the contact point and where the detachment adhesion force was detected.
  All forces measured are relative forces and thus independent of the constant force offset (of 5pN) due to hydrodynamic drag of the force sensor traveling at the constant velocity of 3 µm/s.
- **plateau steps,** for this set of data appear after a force plateau of at least 500 nm length at loading rates of less than 2.7pN/s (see step 2 in **Fig. 3A**).
  At loading rates between 2.7 and 4.0 pN/s the criterion was not clear enough to avoid false positive or negative step discrimination.
- **steep steps** consequently occur after an increase in force of at least 4.0 pN/s.

### Semi-quantitative Polymerase Chain Reaction (PCR)

The semi-quantitative PCR was performed as described in Popov et al, 2011 [26]. Briefly, total RNA was extracted from PC3 and LNCaP cells with RNeasy Mini Kit (Qiagen, Hilden, Germany) and 1 µg RNA was used for cDNA synthesis with AMV First-Strand cDNA Synthesis Kit (Invitrogen). PCR for integrin α1, α2, β1 and GAPDH (used for normalizing the cDNA input) was performed with Taq DNA Polymerase (Invitrogen) in a MGResearch instrument (BioRad, Munich, Germany). Primer sequences and PCR conditions are described in Popov et al, 2011. All PCR results have been reproduced three times independently.

### Statistical Analysis

**Figure 4:**
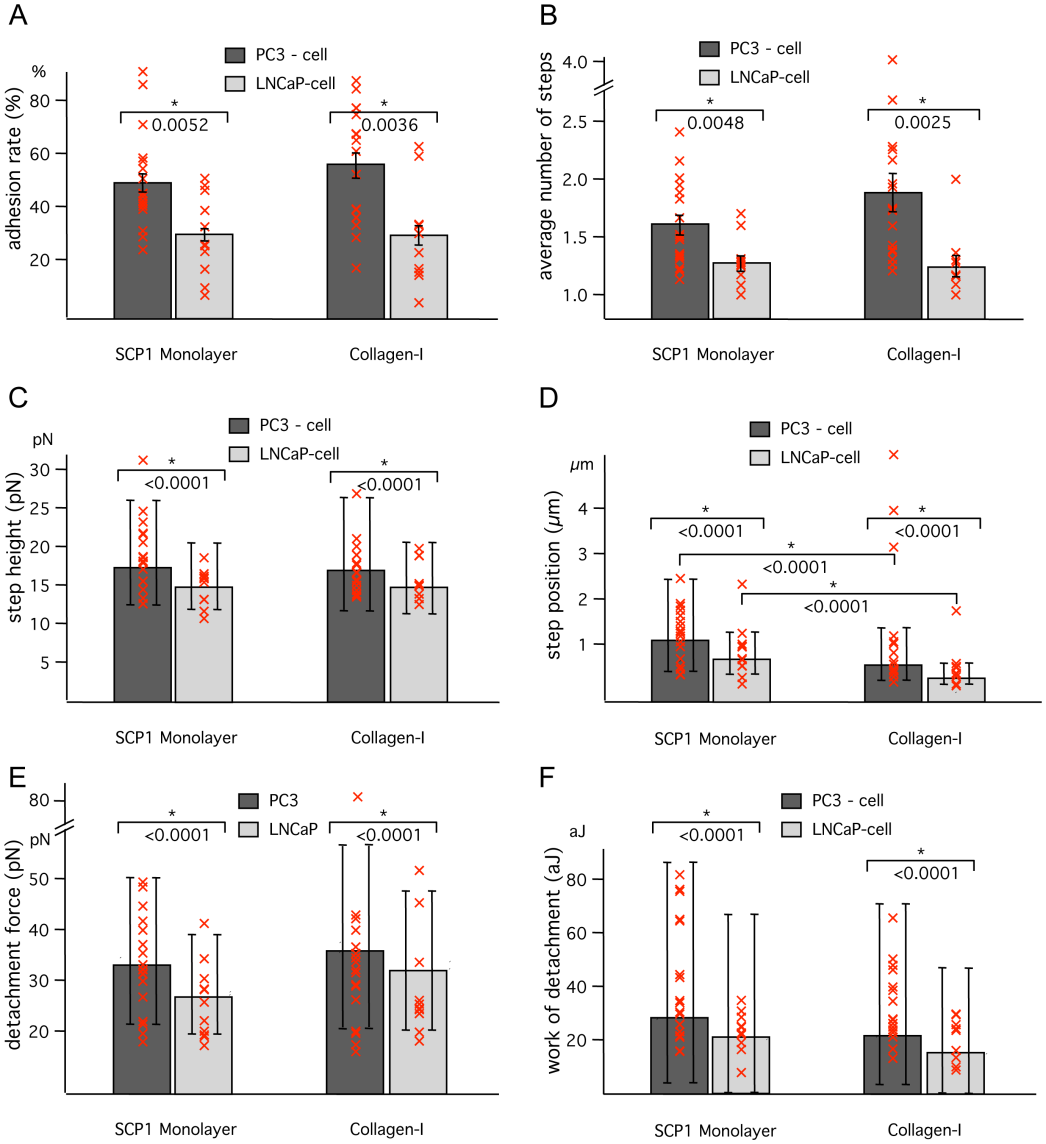
Cell adhesion AFM force spectroscopy measurements of PC cells with Col-I and with SCP1 monolayer. (**A**) Percentages of force curves with at least one de-adhesion event. (**B**) Number of de-adhesion events within one adhesive curve. Error bars correspond to standard error of the mean. A significant p-value from an unpaired t-test of the PC3 data with respect to the LNCaP data is marked by *(p<0.05). The mean of each individual cell is given by a red cross. (**C**) Medians of the height of individual de-adhesion steps. (**D**) Medians of the position of these de-adhesion events. (E) Medians of the detachment force. (F) Medians of the work of detachment. Quartiles are indicated by double flags and the median of each individual cell is given by a red cross. Cell adhesion force data were acquired from 16 PC3 or 10 LNCaP cells interacting with Col-I (1485 and 760 force curves respectively), and 17 PC3 or 11 LNCaP cells interacting with SCP1 monolayers (1526 and 878 force curves respectively).

**Figure 5:**
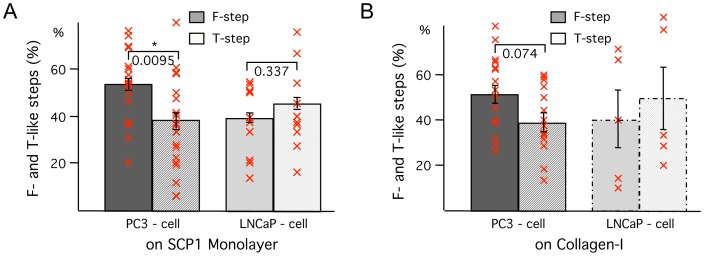
Analysis of filopodia-like steps versus tether-like steps in both cancer cell types to (A) SCP1-monolayers (from more than 600 force curves each) and to (B) collagen-I (from more than 500 PC3 curves but only 54 LNCaP curves; the bars for the LNCaP cells are therefore indicated by dashed lines). Means of the percentage of individual de-adhesion steps representing the typical force pattern of filopodia-like steps (solid) and tether-like steps (striped) for the two cell lines PC3 and LNCaP. Each mean of a cell is represented by a red cross. Error bars correspond to standard error of the mean. A significant p-value from a t-test between the different steps within a prostate carcinoma cell line is indicated by *(p<0.05).

To account for the heterogeneous sets of data two statistical analyses were applied:

First, a Student’s T-test assuming unequal variances was used to analyze the adhesion rate, the average number of steps or the fraction of tether-like to filopodia-like steps comparing the means collected from individual cells between PC3 and LNCaP cells. Each mean of a cell is marked as a red cross; the mean of these means is indicated as bar with error of the mean in **Fig. 4** **A B** and **Fig. 5**. Second, a nonparametric Kolmogorov-Smirnov test without assumptions was applied to compare the step height, step position, detachment force and work of detachment from all force curves between PC3 and LNCaP cells. The medians are indicated as bars with quartiles. Each median of a cell is represented by a red cross in **Fig. 4** **C and D**. Results with a p-value smaller than 0.05 are marked as significant by an asterisk.

## Results

### PC3 and LNCaP Adhesion and Proliferation in Co-culture with SCP1

First cell adhesion was analysed by using time-lapse imaging for up to 12 h. CFDA pre-labelled PC3 and LNCaP cells were monitored on an SCP1 monolayer and after 4 hours, most of the PC3 cells appeared spread on the SCP1 monolayer while the LNCaP cells appeared small and round **(** **Fig. 1A** **)**. As shown in **Fig. S1**, PC3 cells grown on glass or Col-I-coated glass have a lower flatness shape factor compared to LNCaP cells, indicating a higher capacity to spread. However, shape analysis of both cell types cultivated on SCP1 monolayers were not carried out due to the risk of inaccurate measurements of area, diameter and volume due to the underlying cell bodies of the SCP1 cells. Furthermore, by performing quantitative analysis at 4 and 12 h, we could show that approx. 90% of the PC3 cells were able to adhere to the SCP1 monolayer already after 4 h and that their adhesion also remained close to 90% after 12 h **(** **Fig. 1B** **)**. In contrast, LNCaP cells had lower adhesion to SCP1 (approx. 25%), which did not increase significantly after longer cultivation time.

In order to investigate PC3 and LNCaP cell proliferation on SCP1 monolayers, we performed co-culture experiments for up to 8 days. Phase-contrast microscopy at day 1 and 8 demonstrated the formation and propagation of PC3 colonies on top of the SCP1cells, whereas LNCaP cells formed small cell clusters, which did not expand but rather regressed during this period **(** **Fig. 1C** **)**.

Next, the co-cultured cells were counted at three different time points and the growth of PC3 and LNCaP was calculated by subtracting the cell number of SCP1 monolayers cultivated in parallel as controls **(** **Fig. 1D** **)**. Our quantitative analysis confirmed the microscopy observation that PC3 cells but not LNCaP cells were able to divide and further expand on SCP1 cells. In contrast, when cultivated on polystyrene (without SCP1 cells), PC3 and LNCaP cells, have comparable proliferative capacity (**Fig. S2**). Hence, we concluded that PC3 cells have a strong affinity towards SCP1 cells in terms of cell adhesion and proliferation.

### AFM Force Spectroscopy Experiments

To quantify the adhesion forces between prostate cancer cells and the bone matrix protein Col-I as well as the bone marrow-derived mesenchymal stem cell line SCP1 on the single cell level, AFM based force spectroscopy was used [27]–[29].Cell to cell and cell to matrix adhesion experiments were performed in cell culture dishes with cells derived from prostate cancer bone (PC3) or lymph node metastasis (LNCaP). One of these cells was immobilized on the AFM force sensor (**Fig. 2A**), while SCP1 and Col-I were used as substrates in the cell culture dishes. The prostate cancer cell on the AFM cantilever was then brought into contact with Col-I or the SCP1 monolayer for a predefined contact time (0.3 s) and with a predefined contact force (100 pN). Afterwards, the force necessary to withdraw the prostate cancer cell from each of the substrates was recorded. A schematic representation of the experimental setup of the force measurements is depicted in **Fig. 2** (for details, refer to **Materials and Methods**).

The resulting force-distance curves (**Fig. 3**) contain detailed information about the cellular interaction forces on the molecular level [22], [23], [30]. **Fig. 3** shows typical force traces indicating multiple de-adhesion events for PC3 cells **(A, B & C)** compared to single de-adhesion events for LNCaP cells **(D&E)**; on Col-I **(B&D)** and on SCP1 monolayer **(C&E)**. The evaluation of these force curves confirms that PC3 cells exhibit a greater affinity than LNCaP cells to SCP1 cells and Col-I. In order to evaluate these rather complex force-distance curves (**Fig. 3B and 3C**) a step detection algorithm [25] was applied to locate de-adhesion events and to quantify the corresponding forces despite the varying levels of noise. The higher noise levels occurred in experiments on Col-I substrates. This may be due to the undefined anchorage of the glass slide on top of the SCP1 monolayer.

The force measurements of PC3 on SCP1 monolayers showed an overall adhesion rate of more than 45%, whereas the adhesion rate of LNCaP on SCP1 was less than 30%. A similar behavior in adhesion rates was found on Col-I surfaces, where PC3 had an adhesion rate of more than 50% while the adhesion rate of LNCaP was around 30% **(** **Fig. 4A** **)**. These results corroborate previous findings with conventional cell adhesion essays [7], [31]. Also, the average number of de-adhesion force steps from force curves, containing at least one de-adhesion event, is significantly higher for PC3 than for LNCaP, both on SCP1 monolayers and Col-I substrates **(** **Fig. 4B** **)**.

Furthermore, not only the number of adhesive events, but also the forces of the individual de-adhesion steps appeared slightly higher for PC3 cells on both Col-I substrate and SCP1 monolayer, when compared to LNCaP cells **(** **Fig. 4C** **)**.

Because the force step values of the last adhesive event in a force curve did not significantly differ from the values of intermediate steps, all adhesive events were included into the evaluation. Since the force distribution did not follow a Gaussian distribution (except for **Fig. 4A and 4B**), **Fig. 4** depicts medians and quartiles. **Fig. 4C** represents the medians of de-adhesion force steps. For PC3 cells they were at 17,4 pN on SCP1 monolayers and 17.0 pN on Col-I. The step height medians of LNCaP cells, on the other hand, were 14.9 pN on SCP1 monolayers and 14.8 pN on Col-I. Control measurements of PC3 cells on bare glass surfaces incubated with BSA resulted in step forces below 13 pN (not shown).

The same tendency was observed for the detachment force and the work of detachment (**Fig. 4E and 4F**) revealing the PC3 adhesion to SCP1 as the strongest of the four measured interactions and the LNCaP cells as the weaker binders to both Col-I and SCP1. Control measurements on bare glass surfaces incubated with BSA revealed the weakest interactions for all adhesion parameters.

Another parameter, where significant differences were seen between the two prostate cancer cell lines is the step position, i.e. the distance between PC cell and substrate, at which the bond rupture was detected **(** **Fig. 4D** **)**. The adhesive bonds of PC3 cells can be separated from both Col-I substrates and from SCP1 monolayers roughly twice as far as the bonds of LNCaP cells, before they finally break at a median distance of 0,7 µm. The fact that these bonds rupture up to several micrometers away from the observed contact point between the two cell types or between cell and Col-I can be explained by either: a) extremely compliant cells; b) by membrane tethers, which are pulled out of the cell membrane by the external force; or c) by filopodia or other micro-extensions which are actively formed by the cells. Tethers are viscous membrane tubes [12], which are pulled out of the cell membrane at a constant force and therefore exhibit a characteristic force plateau (as shown in **Fig. 3A** before step 2) [15]. Filopodia, on the other hand, are not generated by the pulling force. They contain protruding actin fibers and already exist before the cells are brought into contact with their substrate. Consequently, filopodia are expected to exhibit an initial force-free unbending phase, followed by a sudden increase in force when loaded at a distance from the contact point that corresponds to their initial length. Therefore in contrast to tethers they lack a force plateau. Typical filopodia-like steps can be seen in **Fig. 3B and 3C**. In the case of PC3 cells, more than 50% of all detected steps exhibit these characteristic signatures of filopodia and less than 40% exhibit the typical signature of tethers. For LNCaP cells, on the other hand, less than 40% of the steps appear as filopodia-like steps and about 45% as tether-like steps (**Fig. 5**).

Due to the discrimination criterion, steps at positions shorter than 1 µm were not counted and therefore the ensemble size for LNCaP and on Col-I in particular was small. The number of un-counted steps, because the slope did not allow for a clear distinction between tether and filopodia (loading rates between 2,7 and 4pN/s) was less than 7%. Furthermore, the step position of the filopodia-like steps of PC3 cells increased over time within the experiments at an average rate of 0.6 nm/s, while no significant change in step position was observed in LNCaP cells.

### Integrin Expression in PC3 and LNCaP Cells

**Figure 6:**
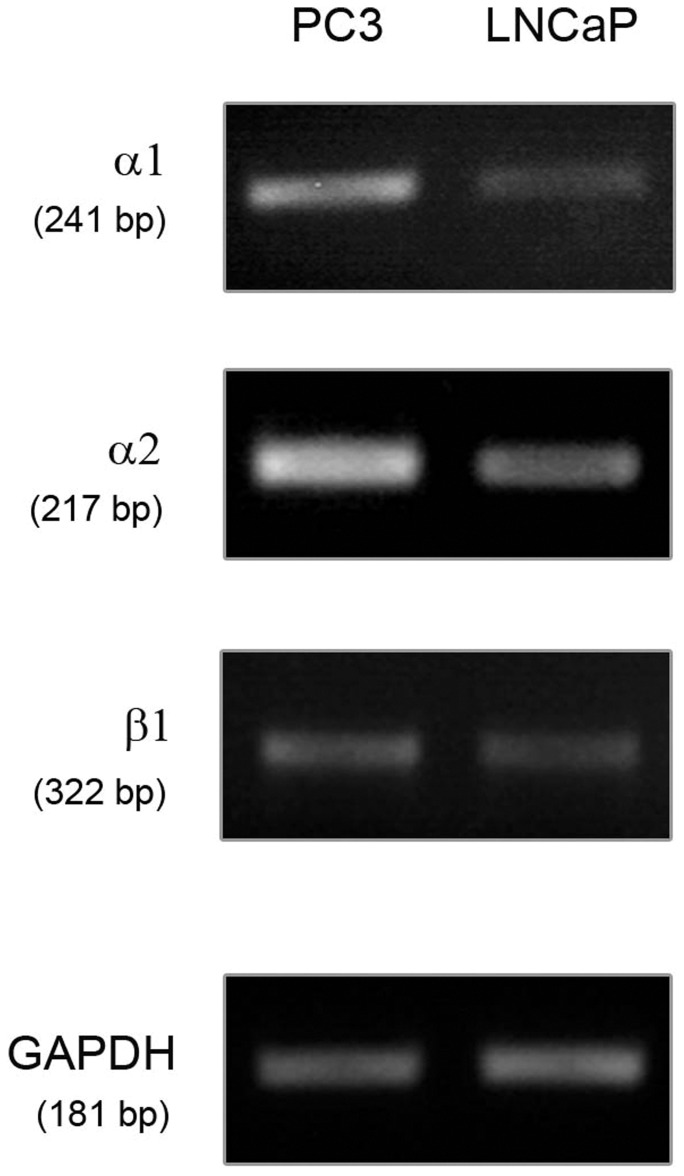
Investigation of integrin expression. Semi-quantitative PCR for α1β1 and α2β1 integrins was performed with cDNA from PC3 and LNCaP cells and revealed a strong expression of both receptors in PC3 cells in comparison to LNCaP cells. The PCR results were reproduced independently three times.

To find out which receptors are possibly responsible for the increased affinity of PC3 cells to collagen type I and SCP1 cells, we investigated the expression of two integrin receptors which have binding affinity to collagen type I, namely α1β1 and α2β1 in PC3 and LNCaP cells by using semi-quantitative PCR. Our results demonstrated that both receptor types are strongly expressed in PC3 cells, in contrast to LNCaP cells (**Fig. 6**).

## Discussion

In this study, the interaction of prostate cancer cells with mesenchymal stem cells and the extracellular matrix protein collagen type I has been investigated both with optical microscopy and with AFM based force spectroscopy. Both approaches highlight different aspects of the cellular interactions between prostate cancer cells and mesenchymal stem cells. Using time lapse optical microscopy, the long-term adhesion was emphasized, starting with initial molecular recognition events and spontaneous adhesion, followed by cellular adaptation processes, such as possible changes in the concentrations of surface receptors caused by cell signaling. Furthermore, a large number of cells could be observed simultaneously using optical microscopy. With the AFM, on the other hand, interaction forces of a much smaller number of cells can be determined quantitatively on the single cell level. This approach concentrates on forces arising during the initial cellular contact, as the cell was not allowed to develop the cell contact for more than 0.3 seconds before it was retracted and forced to unbind.

Previous studies using optical microscopy as well as AFM imaging already showed that PC3 cells adhere and proliferate much better than LNCaP cells on Col-I and that PC3 adhesion, proliferation, and cell stiffness is significantly enhanced on Col-I, compared to other ECM proteins, such as fibronectin [7]. The time lapse microscopy results presented here show a similar behavior of PC3 cells co-cultured together with the mesenchymal stem cell line SCP1. From the first hours of co-cultivation up to several days in culture, prostate cancer cells derived from bone metastasis (PC3) proliferate and spread well on MSCs, while the control group, which was derived from lymph metastasis (LNCaP), not only shows much fewer adherent cells during the initial hours of co-cultivation, but the number of cells even decreased after five days in culture.

To obtain a deeper insight into the nature of the observed cell-cell and the previously described cell-matrix interactions, we quantified the interaction forces on the single cell level using AFM based force spectroscopy. Although, as discussed above, AFM only probes the initial cellular contacts, the results agree with the optical microscopy results, as well as with the previous findings [7]. On both Col-I and on the SCP1 monolayer, the percentage of cellular interactions (adhesion rate), the number of interactions per successful force experiment (number of steps), the step position, the force of a single interaction event (step height), the detachment force, and the total work of detachment were larger for SPC3 than for LNCaP. Furthermore, although the force curves varied statistically in shape and complexity, except for the slow increase in rupture position of filopodia-like ruptures in PC3 cells, we could not observe any systematic changes in the adhesion pattern or signs of spreading during repeated force measurements on a single cell. This could explain why the results from both the long term observations with optical microscopy and short term AFM based force spectroscopy show a similar picture: the set of adhesion molecules present on the prostate cancer cells appears to be constant from the initial contact to the following hours and possibly even days and sufficient to determine their metastatic behavior.

Our findings clearly demonstrated that PC3 cells are very distinct from LNCaP cells in regards to their adhesive behavior. In particular, PC3 cells showed significantly stronger adhesion on both substrates when compared to LNCaP. Furthermore, within each individual cell line, PC3 or LNCaP, both cell types did not show statistically significant differences in the parameter values extracted from the AFM measurements on Col-I and on SCP1. Except for the step position that was shorter on Col-I and larger on SCP-1 monolayers due to the fact that the cells of the monolayer contributed their compliance and membrane tethers to enlarge the interaction distances. These findings indicate that the adhesion of the prostate cancer cells to mesenchymal stem cells could be mediated mainly by their interaction with Col-I, which is expressed extracellularly by MSCs [32]. This observation was confirmed by immunofluorescence staining of Col–I in SCP1 cells and on the Col–I coated microscope slides, which both showed a strong fluorescence signal (see inset in **Fig. 2A**). Consistent with previous reports showing that PC3 cells express a number of Col-I binding integrin receptors [31] while LNCaP cells lack some of these integrins [33] we show that α1β1 and α2β1 integrins are potential candidates to mediate the detected force patterns.

Finally, our observation that PC3 cells exhibit rupture events at much more extended positions than LNCaP cells (**Fig. 3** **and** **4D**) and that the extensions grow during the experiment may reflect the fact that PC3 cells tend to actively extrude filopodia when they come into contact with Col-I, while LNCaP cells keep their defined smooth surface. This observation is consistent with high resolution AFM and fluorescence microscopy studies [7], which showed that on Col-I coated substrates, PC3 cells exhibit a large number of well pronounced filopodia, while LNCaP cells on Col-I coated substrates remain smooth and show almost no filopodia.

### Conclusions

We have shown that prostate cancer cells derived from bone metastasis (PC3) have a higher affinity to mesenchymal stem cells (SCP1 cell line) as well as to the extra cellular bone matrix protein collagen type I (Col-I), than lymph-derived prostate cancer cells (LNCaP). On both substrates, PC3 show enhanced proliferation and spreading, as well as more frequent interactions and stronger adhesion forces and energies. The Col-I staining experiments and the similarities between the cellular de-adhesion events of PC3 on SCP1 cells and on Col-I point to Col-I binding receptors such as α1β1 and α2β1 integrins being largely responsible for the measured interactions. Further experiments with specific integrin knock-down cells, as well as a quantitative analysis of the expression levels of these receptors will help to identify the responsible adhesion molecules. This approach may help to elucidate the mechanisms responsible for prostate cancer metastasis in bone and possibly identify new targets for anticancer drugs in the future.

## Supporting Information

Figure S1 **Flatness shape factor of PC3 and LNCaP cells, cultivated on glass or Col-I coated glass slides, was calculated as described in Docheva et al** [**7**] **.** The results revealed that PC3 cells are flatter on both surfaces compared to LNCaP cells. Graph bars represent mean ± SD of at least three independent AFM scans for both cell type on each surface.
(TIF)

Figure S2 **Analysis of PC3 and LNCaP proliferation on polystyrene.** Both cell types were cultivated in T-75 flasks and during passaging over a period of 24 days their number was recorded. Cumulative population doubling (cum PD) and population doubling time (PDT) were calculated as described in Huang GT et al 2006 [34]. The obtained results demonstrate that in a non co-culture condition both cell types have comparable proliferative capacity. In the calculation of PDT, graph bars represent mean ± SD of the different passages for each cell type.
(TIF)
