# Correction: Probing the Interaction Forces of Prostate Cancer Cells with Collagen I and Bone Marrow Derived Stem Cells on the Single Cell Level

**DOI:** 10.1371/annotation/d7f2081e-8756-4253-a066-7fb084cd0759

**Published:** 2013-10-17

**Authors:** Ediz Sariisik, Denitsa Docheva, Daniela Padula, Cvetan Popov, Jan Opfer, Matthias Schieker, Hauke Clausen-Schaumann, Martin Benoit

The loading rates in the "Cell Adhesion Force Evaluation" section of the Material and Methods section are incorrect.

The correct parameters h) and i) should be:

h) **plateau steps**, for this set of data appear after a force plateau of at least 500 nm length at

loading rates of less than 27pN/s (see step 2 in Fig. 3A).

At loading rates between 27 and 40 pN/s the criterion was not clear enough to avoid false positive or negative step discrimination.

i) **steep steps** consequently occur after an increase in force of at least 40 pN/s.

The loading rates in the Results above the "Integrin Expression in PC3 and LNCaP Cells" section are incorrect. The corrected loading rates are: (loading rates between 27 and 40 pN/s ) was less than 7%.

