# Supplementary figures and images for: Probing the Interaction Forces of Prostate Cancer Cells with Collagen I and Bone Marrow Derived Stem Cells on the Single Cell Level
Source: PLoS One. 2013 Mar 5;8(3):e57706. doi: 10.1371/journal.pone.0057706 (PMC3589411; doi:10.1371/journal.pone.0057706)

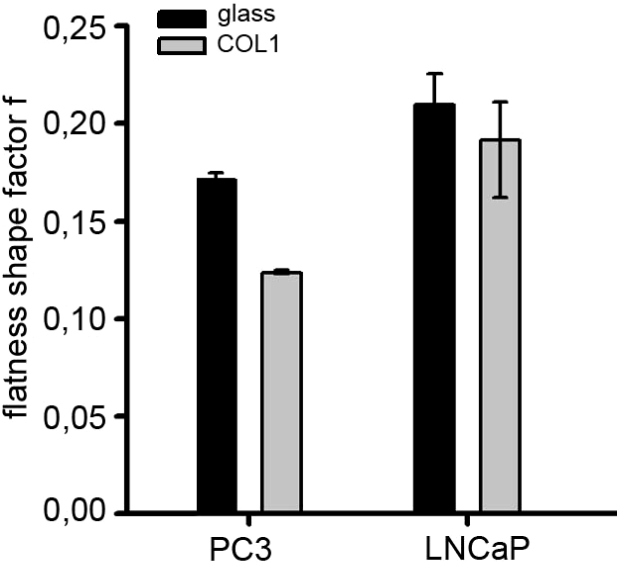

Supplement: Figure S1 — Flatness shape factor of PC3 and LNCaP cells, cultivated on glass or Col-I coated glass slides, was calculated as described in Docheva et al [7] . The results revealed that PC3 cells are flatter on both surfaces compared to LNCaP cells. Graph bars represent mean ± SD of at least three independent AFM scans for both cell type on each surface. (TIF) [file pone.0057706.s001.tif]

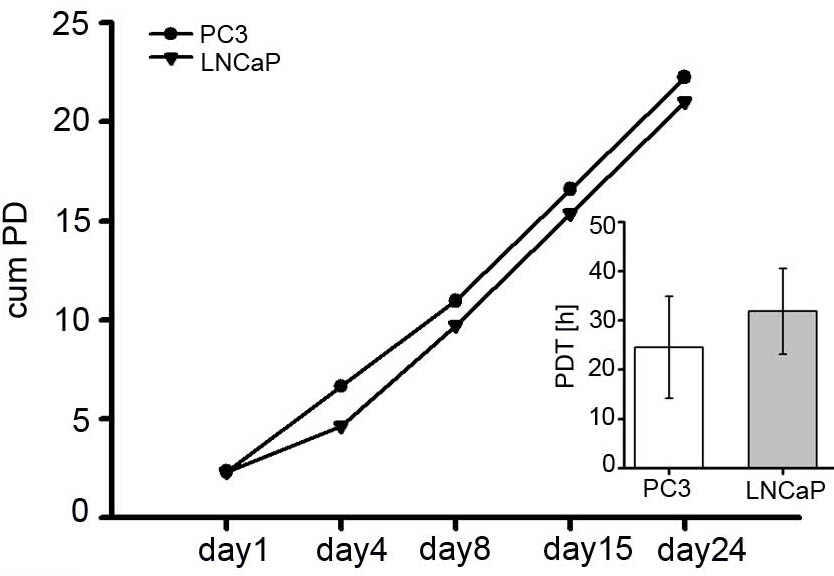

Supplement: Figure S2 — Analysis of PC3 and LNCaP proliferation on polystyrene. Both cell types were cultivated in T-75 flasks and during passaging over a period of 24 days their number was recorded. Cumulative population doubling (cum PD) and population doubling time (PDT) were calculated as described in Huang GT et al 2006 [34]. The obtained results demonstrate that in a non co-culture condition both cell types have comparable proliferative capacity. In the calculation of PDT, graph bars represent mean ± SD of the different passages for each cell type. (TIF) [file pone.0057706.s002.tif]
